# A new species of the *Boophis rappiodes* group (Anura, Mantellidae) from the Sahamalaza Peninsula, northwest Madagascar, with acoustic monitoring of its nocturnal calling activity

**DOI:** 10.3897/zookeys.435.7383

**Published:** 2014-08-18

**Authors:** Samuel G. Penny, Franco Andreone, Angelica Crottini, Marc W. Holderied, Lovasoa Sylviane Rakotozafy, Christoph Schwitzer, Gonçalo M. Rosa

**Affiliations:** 1 School of Biological Sciences, Life Sciences Building, University of Bristol, Tyndall Avenue, Bristol, BS8 1TQ, UK; 2 Bristol Zoological Society, c/o Bristol Zoo Gardens, Clifton, Bristol, BS8 3HA, UK; 3 Museo Regionale di Scienze Naturali, Via G. Giolitti, 36, I-10123, Torino, Italy; 4 CIBIO Research Centre in Biodiversity and Genetic Resources, InBIO, Universidade do Porto, Campus Agrário de Vairão, Rua Padre Armando Quintas, Nº 7, 4485-661 Vairão, Vila do Conde, Portugal; 5 Département de Biologie Animale, Faculté des Sciences, Université d’Antananarivo, BP 496, Antananarivo (101), Madagascar; 6 Durrell Institute of Conservation and Ecology, School of Anthropology and Conservation, University of Kent, Canterbury, Kent, CT2 7NR, UK; 7 Institute of Zoology, Zoological Society of London, Regent’s Park, NW1 4RY London, UK; 8 Centro de Biologia Ambiental, Faculdade de Ciências da Universidade de Lisboa, Bloco C2, Campo Grande, 1749-016 Lisboa, Portugal

**Keywords:** Amphibia, *Boophis ankarafensis* sp. n., Sahamalaza – Iles Radama National Park, advertisement call, conservation

## Abstract

A new species of treefrog of the *Boophis rappiodes* group (Anura, Mantellidae) is described from the Sahamalaza – Iles Radama National Park in northwest Madagascar. This new species is green in colour with bright red speckling across its head and dorsum; similar in morphology to other species of this group including: *B. bottae*, *B. rappiodes*, *B. erythrodactylus* and *B. tasymena*. The new species can be distinguished by its advertisement call and by a genetic divergence of more than 4.9% in the analysed mitochondrial 16S rRNA gene fragment. Its call consists of two note types: a trill and a click; although similar sounding to *B. bottae*, the trill note of the new species has a faster pulse rate while the click note is predominantly two-pulsed rather than three. All individuals were detected from the banks of two streams in Ankarafa Forest. The new species represents the only member of the *B. rappiodes* group endemic to Madagascar’s western coast, with the majority of other members known from the eastern rainforest belt. Despite its conspicuous call, it has not been detected from other surveys of northwest Madagascar and it is likely to be a local endemic to the peninsula. The ranges of two other amphibian species also appear restricted to Sahamalaza, and so the area seems to support a high level of endemicity. Although occurring inside a National Park, this species is highly threatened by the continuing decline in the quality and extent of its habitat. Due to these threats it is proposed that this species should be classified as Critically Endangered according to the IUCN Red List criteria.

## Introduction

The genus *Boophis* is a monophyletic group of treefrogs belonging to the family Mantellidae. Endemic to Madagascar and the Comoros, the group comprises over 70 species, many of which have only recently been described (Köhler et al. 2007, 2008, 2011; Wollenberg et al. 2008; Glaw et al. 2010; Vallan et al. 2010; Vences et al. 2010, 2012). The genus is classified into two subgenera, *Boophis* and *Sahona*, which largely correlate with breeding habit; the nominal *Boophis* being predominantly stream-breeders, and the *Sahona* pond-breeders (Glaw and Vences 2006). Of the two subgenera, *Boophis* is the most speciose and can be further divided into eight groups, although at least some of these appear to be paraphyletic and await taxonomic revision (Glaw et al. 2010). Among these is the *rappiodes* group – small, green treefrogs with red pigment patches and partially translucent skin – an appearance shared with the neotropical glass frogs of the family Centrolenidae (Vences and Glaw 2002; Glaw and Vences 2007; Köhler et al. 2007). As with most Malagasy amphibian diversity, the majority of members of the *rappiodes* group can be found in the eastern rainforests, and until now there have been no documented species from the drier forests of the western coast (Vences and Glaw 2002; Glaw and Vences 2007).

The Sahamalaza Peninsula in north-western Madagascar has undergone only two previous amphibian surveys (Andreone et al. 2001; Raselimanana 2008); the most recent of which led to the discovery of *Boophis tsilomaro* and *Cophyla berara* (Vences et al. 2005, 2010). However, one site on the peninsula, the Ankarafa Forest, had never been surveyed before. From this forest we describe a new species of *Boophis*, molecularly assigned to the *rappiodes* group, vocally distinct and genetically different to all other known species of this monophyletic group.

## Methods

### Study sites and survey periods

**Figure 1.**
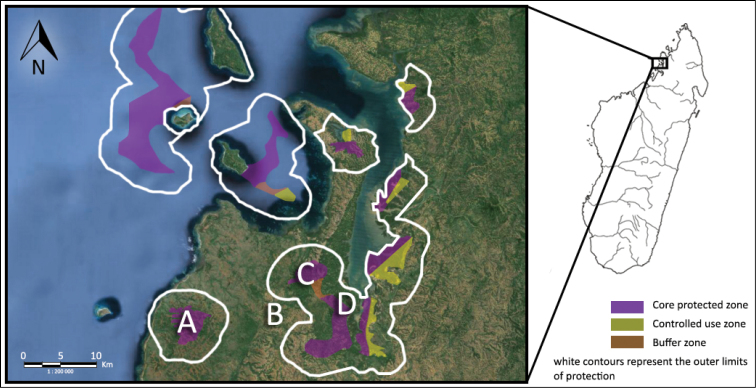
The Sahamalaza Peninsula in northwest Madagascar, indicating the study sites of (**A**) Ankarafa Forest, (**B**) Antafiabe Village, (**C**) Anabohazo Forest and (**D**) Betsimpoaka village. Source: Madagascar National Parks (MNP).

The Sahamalaza Peninsula is in the province of Mahajanga, northwest Madagascar (Fig. 1). Parts of the peninsula were declared the Sahamalaza – Iles Radama National Park in July 2007 and have been included in UNESCO’s network of Biosphere Reserves since 2001 (Schwitzer et al. 2007). The peninsula covers around 26,000 ha and is characterised by a number of low hills of about 300-350 m a.s.l. intersected by a few largely seasonal streams (Andreone et al. 2001). Located between 13°52'S and 14°27'S, and 45°38'E and 47°46'E, it is defined by the Sahamalaza Bay to the east, the Mozambique Channel to the west and the Loza River to the south (Volampeno 2009).

The climate is sub-humid and has two distinct seasons: a cooler, drier season from May to November; and a hotter, wetter season from December to April. Monthly mean maximum temperature ranges from 28.5 ± 3.61 °C in July to 39.1 ± 2.11 °C in February; and monthly mean minimum temperature ranges from 13.2 ± 0.81 °C in October to 21.8 ± 0.81 °C in January (Volampeno et al. 2011). Rainfall is highest during January and February and the mean annual precipitation rate is around 1,600 mm (Schwitzer et al. 2007).

Sahamalaza supports a unique type of transitional forest that harbours plant species from both the wetter Sambirano domain and drier western domain (Birkinshaw 2004; Schwitzer et al. 2006). This forest is concentrated in two separate blocks: Anabohazo in the northeast (14°18.56'S, 47°54.89'E) and Ankarafa in the west (14°22.82'S, 47°45.46'E) (Schwitzer et al. 2007). Until recently a third block existed, called Analavory Forest (14°23.30'S, 47°56.15'E), but it experienced near complete destruction following an uncontrolled man-made fire in 2004 (Volampeno 2009). The forest blocks are composed of a matrix of smaller fragments isolated by savannah with high levels of human disturbance (Schwitzer et al. 2007).

Fieldwork took place over two separate periods: a three month survey between October 2011 and January 2012, and an additional survey between January and February 2013. This ensured the coverage of most of the wet season when rain is more abundant and the individuals are expected to be more active. Surveys were conducted in Ankarafa Forest, Anabohazo Forest and around the villages of Antafiabe and Betsimpoaka.

### Survey methods

Surveys were conducted by opportunistic searching and directed towards vocalising males using headlamps and torches. Transects were made across a range of habitats and degradation levels, covering the four previously mentioned areas of the peninsula. Searches were repeated during the day and night to account for any diel differences in activity, taking place in the morning and evening. Searching took place approximately two metres either side of the transect and up to two metres in height. Most searches in Ankarafa were repeated at least once both in the dry and wet season (during the 2011–2012 period) following the same routes where possible. The sites in Anabohazo Forest, Antafiabe and Betsimpoaka village were sampled only once. Sites were sampled in a randomised order and all searches were conducted by the same two individuals to avoid systematic observer bias. The frogs’ frequency of occurrence was estimated by dividing the total number of frogs encountered during each transect search by the respective length.

The vouchers were photographed to document life colouration and calls were recorded whenever possible. Acoustic description follows that outlined in the acoustic analysis methods below. The tissue samples (fourth digit of the left toe removed with scissors) were stored in 70% ethanol or 96% ethanol for genetic analysis. Location was logged using a handheld GPS receiver (Garmin eTrex Vista HCx; Garmin International Inc., Olathe, United States). Microhabitat was noted and vertical position from the ground measured using a tape measure.

### Morphological measurements

Specimens were collected both day and night, euthanized in a chlorobutanol solution, fixed in 90% ethanol or 5% formalin, and preserved in 70% ethanol. Specimens are deposited in the collections of Museo Regionale di Scienze Naturali, Torino, Italy (MRSN; Table 1). Morphological measurements (in millimetres) were taken with a digital calliper (precision 0.01 mm) to the nearest 0.1 mm by F.A. Used abbreviations are: SVL (snout-vent length), HW (greatest head width), HL (head length), ED (horizontal eye diameter), END (eye-nostril distance), NSD (nostril-snout tip distance), NND (nostril-nostril distance), TD (horizontal tympanum diameter), TL (tibia length), HAL (hand length), FOL (foot length), FOTL (foot length including tarsus), FORL (forelimb length), HIL (hindlimb length), RHL (reaching of tibiotarsal articulation when hindlimb is adpressed along body). Terminology and description follows Vences and Glaw (2002) and Glaw and Vences (1997) for eye colouration. Webbing formulae follow Blommers-Schlösser (1979).

**Table 1.** Morphometric measurements (in mm) of preserved specimens of *Boophis ankarafensis* sp. n. For abbreviations of variables, see methods.

MRSN	A6973	A6974	A6975	A6976
STATUS	HOLOTYPE	PARATYPE	PARATYPE	PARATYPE
SEX	male	female	male	male
LIFE STAGE	adult	adult	adult	adult
SVL	24.0	28.5	23.7	22.9
HW	9.1	12.0	8.4	8.5
HL	7.8	11.5	8.2	8.1
ED	4.1	4.7	3.6	3.6
END	2.1	3.3	2.6	2.7
NSD	2.6	2.8	2.2	2.0
NND	3.0	3.8	2.7	2.3
TD	1.5	1.6	1.7	1.2
TL	12.7	17.2	12.0	10.8
HAL	8.1	9.2	7.5	6.1
FOL	11.2	14.1	10.2	9.6
FOTL	17.2	22.8	16.4	15.6
FORL	15.1	21.3	14.1	11.5
HIL	41.1	53.7	37.9	37.5

### DNA analysis

Tissue samples were available for four individuals. Total genomic DNA was extracted from the tissue samples using proteinase K digestion (10 mg/ml concentration) followed by a standard salt-extraction protocol (Bruford et al. 1992). We sequenced a fragment of ca. 550 bp of the 3' terminus of the mitochondrial 16S rRNA gene. For primers and cycling protocols see Crottini et al. (2011). The light strands were sequenced using an ABI3730XL by Macrogen Inc.

Sequences were checked by eye, edited and aligned using the BioEdit sequence alignment editor (version 7.0.5.3; Hall 1999). The alignment of the four processed samples and of the other species belonging to the *Boophis rappiodes* group taken from GenBank (12 homologous sequences of *Boophis bottae* from Ranomafana (8), Andasibe (2) and Betampona (2); 10 homologous sequences of *Boophis rappiodes*, from Ranomafana (5) and Andasibe (5); 1 homologous sequence of *Boophis erythrodactylus*, from Mandraka; 22 homologous sequences of *Boophis tasymena*, from Andasibe (1), An’Ala (5), Ranomafana (15) and Maharira (1); and 18 homologous sequences of *viridis*, from Ranomafana (1), Andasibe region (16) and Betampona (1)) required the inclusion of gaps to account for indels in only a few cases in some hypervariable regions. All newly determined sequences have been deposited in GenBank (KJ438141–KJ438144).

To assess genetic distinctness of the new species from all other Malagasy frogs and ascertain its belonging to the *Boophis rappiodes* group, sequences were compared using the BLAST algorithm with a database containing homologous sequences of reliably identified adult individuals of almost all Malagasy frog species (Vieites et al. 2009). Mean genetic distances matrix (uncorrected *p*-distance transformed into percent, using the complete deletion option) between and within individuals belonging to the type series of *Boophis ankarafensis* sp. n. (holotype and three paratypes) and of other species of the *Boophis rappiodes* group were computed using MEGA, version 6.06 (Tamura et al. 2013).

### Acoustic monitoring and analysis

Most amphibian calls are species-specific and it is usually possible to identify syntopic calls to the species level. Sound recordings were taken to obtain detailed information on the acoustic parameters of the species and investigate any intraspecific variability and overnight temporal patterns in activity. Acoustic recordings were made continuously from dusk until dawn on sixty nights between October 2011 and January 2012. Data were collected from 37 different locations; the majority of these locations were within Ankarafa Forest (29), followed by Anabohazo Forest (7) and a single location on the Vavan’aneno River near the village of Antafiabe. Nineteen locations had recordings made on two or more nights, separated between 9 and 79 days.

Calls were recorded in the field using a Song Meter SM2 digital recorder (Wildlife Acoustics Inc, Concord, United States) at a 16-bit resolution and 16 kHz sampling rate using two side-mounted SMX-II microphones. The digital recorder was placed one to two metres above the ground or water by securing it to deadwood or a protruding branch using bungee cords. Continuous recordings split into sections of 120 minutes each were saved in the standard uncompressed .WAV format. Preceding analysis, these were split using a custom-written MATLAB (The Mathworks, Natick, USA, v7.14.0.739) script into minute long segments to allow for more efficient analysis. Spectrograms were viewed individually as a dual channel output using Avisoft SASlab Pro (Berlin, Germany, v5.2.06); frequency resolution of 512 FFT, a 100% frame rate, Hamming window and an intensity threshold of 50%.

From each night-long recording where *Boophis ankarafensis* sp. n. was detected, five representative vocalisations for each identified call type were chosen. Calls with the highest recording quality were selected and where possible equally distributed over the entire recording period. This reduced the risk of selecting the same individual repeatedly (pseudoreplication) by maximising the time period between each of the selected vocalisations. From a subset of five trill notes, acoustic parameters were measured for ten sequential broadband pulses and ten sequential narrowband pulses per note.

From each of these notes spectral and temporal characteristics were measured, and the minimum, maximum and average values (with SD) calculated (Avisoft SASlab Pro; Berlin, Germany; v5.2.06). To remove interspecific and abiotic noise outside of the *Boophis ankarafensis* sp. n. bandwidth, and increase detection thresholds, sound files were band-pass filtered with a finite impulse response filter (Hamming) between 3.2 and 6 kHz using Avisoft SASlab Pro (Berlin, Germany, v5.2.06). The spectral measurements taken were peak frequency (= frequency of maximum energy) and bandwidth. These were calculated using averaged power spectra with peak interpolation over three data points (Hamming window, FFT width 512 points; bandwidth threshold -10dB; peak detection threshold -20 dB, hysteresis 10 dB). For the trill note (type 1) these were taken from each of the two pulse types present and also a section of 40–60 sequential pulses; measurements were taken from the click note (type 2) in its entirety. Durations were taken for the two notes types in their entirety and also for individual pulses. The pulse rate of the trill note was calculated by taking the inverse of the average time differences between pulses, calculated through pulse train analysis. All measurements are stated as the mean ± standard deviation followed in parentheses by the maximum and minimum and number of analysed units (n). Calls were then compared to an existing database of frog vocalisations (Vences et al. 2006; Rosa et al. 2011): comparisons were limited to notes recorded at similar ambient temperatures as, to a limited extent, some call parameters can be temperature dependent (Glaw and Vences 2007).

Minute by minute changes in activity were recorded for all detected species from dusk (sunset) until dawn (sunrise) for 60 nights. The activity index per minute was 1 for one caller and 2 for more than one. The time period between dusk and dawn was split into percentiles for each night; this controlled for the slight variations in night length experienced across the sampling period, enabling the summation of data across multiple nights for each analogous percentile. The number of minute periods containing calling activity within each percentile was calculated and the values for each analogous percentile across all nights summed. The totals for each percentile were then divided by the total activity to determine the proportional change in activity.

## Results

### Boophis ankarafensis sp. n.

Taxon classificationAnimaliaAnuraMantellidae

http://zoobank.org/65D4E091-5D71-4323-963D-5494D6460EE1

#### Etymology.

The term *ankarafensis* is a specific epithet deriving from the species’ *terra typica*, the Ankarafa Forest. The name is used as an adjective in the nominative singular.

#### Holotype.

**Figure 2.**
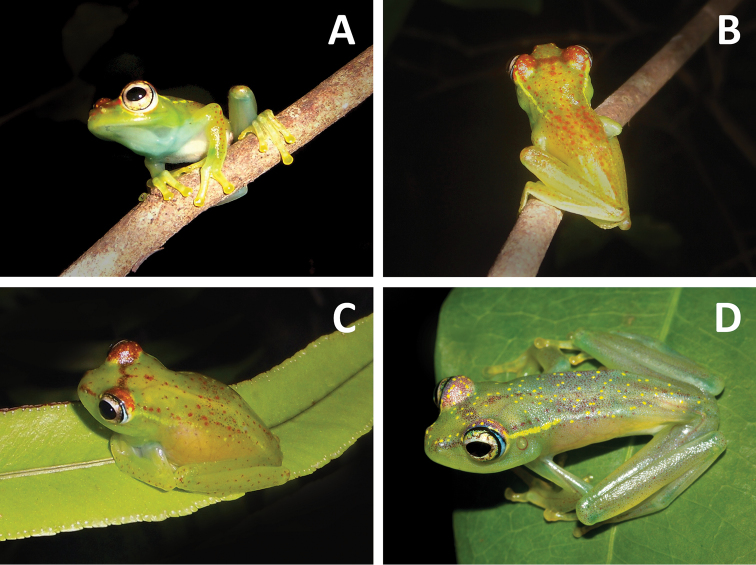
Life colouration of *Boophis ankarafensis* sp. n.: **A** Rostral view of a male paratype (MRSN A6975) **B** Dorsal view of the same male **C** Female specimen in resting position on a leaf (specimen not collected) **D** Dorso-lateral view of the holotype with day-time colouration (MRSN A6973).

**Figure 3.**
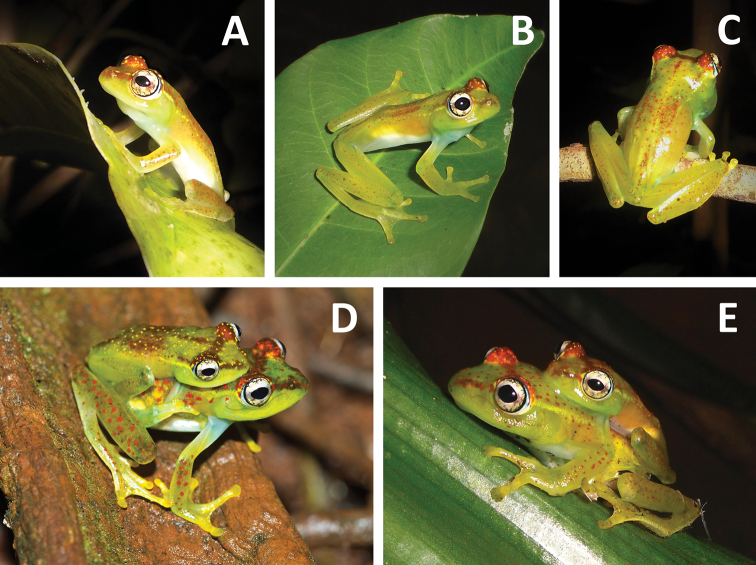
Breeding activity of *Boophis ankarafensis* sp. n.: **A** Paratype MRSN A6976 **B–C** Vocalising males sitting on leaves and on a branch (specimens not collected) **D** Male holotype MRSN A6973 and female A6974 **E** Couple in axillary amplexus.

MRSN A6973, adult male (Fig. 2D and Fig. 3D) collected at Ankarafa Forest (Sahamalaza Peninsula, north-western Madagascar), 14°22.85'S, 47°45.52'E; ca 140 m a.s.l., transitional forest, 26 January 2013, leg. G. M. Rosa.

#### Paratypes.

MRSN A6974 adult female (Fig. 3D), same data as holotype. MRSN A6975, adult male (Fig. 2A–B) collected at Ankarafa Forest (Sahamalaza Peninsula, north-western Madagascar), 14°22.83'S, 47°45.47'E; ca 130 m a.s.l.; transitional forest, 21 November 2011, leg. S. G. Penny. MRSN A6976, adult male (Fig. 3A) collected at Ankarafa Forest (Sahamalaza Peninsula, north-western Madagascar), 14°22.85'S, 47°45.51'E; ca 130 m a.s.l., transitional forest, 12 January 2012, leg. S. G. Penny.

#### Diagnosis.

A treefrog assigned to the genus *Boophis* based on absence of femoral glands in males, the presence of an intercalary element between the ultimate and penultimate phalanges of fingers and toes (verified by external examination), presence of nuptial pads in males, general morphological resemblance to other *Boophis* species, and molecular evidence. Assigned to the *Boophis rappiodes* group based on small size (adult males 22.9–24.0 mm and one female 28.5 mm SVL), absence of lateral fringes along lower arm and tarsus, greenish and slightly translucent dorsal colouration and translucent venter (inner organs can be clearly seen through the skin in live specimens). *Boophis ankarafensis* sp. n. is distinguished from *Boophis erythrodactylus* by lack of reddish colour of fingertips, and from *Boophis erythrodactylus* and *Boophis tasymena* by lack of regular pattern of red dorsal spots. Distinguished from *Boophis viridis* by its smaller size (SVL up to 31 mm in males and 35 mm in females of *Boophis viridis*), iris colouration (*Boophis viridis* shows a distinctive brown inner-iris area and blue outer-iris area), and the presence (vs. absence) of yellowish dorsolateral stripes. Compared to *Boophis rappiodes*, the new species is distinguished by a more extensive darker pattern on the dorsum, which is most evident in living or freshly preserved specimens; while the dorsal pattern in *Boophis rappiodes* is intensely red, and remains red in preservative before it eventually fades, the pattern in *Boophis ankarafensis* sp. n. is reddish-brown in life and becomes persistently dark brown in preservative, often covering almost the entire dorsum. *Boophis ankarafensis* sp. n. is most similar in morphology to *Boophis bottae* and no apparent morphological feature distinguishes them. However, the new species can be distinguished by molecular analysis and by its advertisement calls (see below).

#### Description of the holotype.

MRSN A6973, adult male in a good state of preservation. SVL 24.0 mm (see Table 1 for more detailed morphometric measurements). The body is slender with the head much wider than the body. Snout rounded in dorsal view, slightly truncate in lateral view, nostrils directed laterally, slightly nearer to tip of snout than to eye; canthus rostralis and loreal region both slightly concave; tympanum distinct, rounded, 40% of eye diameter; supratympanic fold not recognizable; tongue ovoid, distinctly bifid, posteriorly half free; vomerine odontophores distinct; positioned posteromedian to choanae; choanae small, rounded. Arms slender, subarticular tubercles single, round; metacarpal tubercles unrecognizable; fingers with weak webbing; webbing formula 1(1), 2i(1.75), 2e(0.75), 3i(2.5), 3e(1.75), 4(1); relative length of fingers 1 < 2 < 4 < 3 (finger 2 distinctly shorter than finger 4); finger disks moderately enlarged; small unpigmented nuptial pads faintly recognizable on inner side of first finger. Hindlimbs slender; tibiotarsal articulation reaches nostril when hindlimb is adpressed along body; lateral metatarsalia separated by webbing; inner metatarsal tubercle distinct, no outer metatarsal tubercle; webbing formula between toes 1(0), 2i(0.75), 2e(0), 3i(0.75), 3e(0), 4i(1), 4e(1.5), 5(0.25); relative length of toes 1 < 2 < 5 = 3 < 4; toe disks slightly enlarged. Skin on dorsal surfaces and ventrally on throat smooth; ventral skin on belly and around cloacal opening glandular. The ground dorsal colour, including limbs, is light green. The webbing, finger and toe disks are green-yellow in colour. Speckles of reddish-brown and yellow pigment can be seen covering the dorsum and limbs. Thin yellow dorsolateral stripes run from behind the eye to the forelimb and then fade towards the mid-body. On the head a reddish-brown pigment forms a band between the eyes and covers the supra-ocular area, interspersed with yellow speckling. This reddish-brown pigment also forms a faint rostral stripe between the eye and nose tip. The pupil is horizontal, with a beige iris containing darker brown patches and reticulations; the iris periphery is blue. The specimen has a white venter with some translucence exposing the inner organs, and a bluish throat. At day-time the green on the dorsum became pale and the red markings fade out to a pale brownish red colour (Fig. 2D). In preservative it is in good condition and comparatively more hydrated. The colouration, at about 4 months from the capture is vivid: the dorsum is pigmented and the dark bar between the eyes is contrasting and evident, as well as the pigmentation around the nostrils. The specimen has the last phalanx of the 4th toe of the left foot missing for genetic analysis.

#### Description of paratypes and variation.

The paratypes (MRSN A6974-6976) closely match the holotype but with slightly different patterning of pigment patches. Finer regular black spots were observed on the dorsum and limbs (possibly single melanophores); a feature similarly observed in the related *Boophis bottae* (Vences and Glaw 2002). Based on other individuals photographed in nature, specimens matched the holotype and paratypes. However, pigment patterns vary in their distribution and intensity. The males MRSN A6975 and MRSN A6976 are in mediocre state of conservation and dehydrated due to fixation and preservation in ethanol. The colouration, after about 18 months from the capture, faded from green to whitish-yellowish except for pigmentation on the upper eyelids and for the bar between the eyes. The dorsum and upper parts of legs are finely pigmented. Eyes are blackish, with whitish pupillae. The 5th toe of the left foot was removed and fixed in 96% ethanol for molecular analyses. The female specimen (SVL 28.5 mm) collected more recently (MRSN A6974) is in better condition, similar to the holotype. This specimen also has the last phalanx of the 4th toe of the left foot missing for molecular identification. See Table 1 for detailed morphometric measurements.

#### Mitochondrial variation and differentiation.

The molecular data confirms the attribution of *Boophis ankarafensis* sp. n. to the *Boophis rappiodes* group (Glaw and Vences 2007). The four analysed specimens of *Boophis ankarafensis* are genetically uniform and did not show any intraspecific divergence, while the analysed specimens of *Boophis bottae*, *Boophis rappiodes*, *Boophis tasymena* and *Boophis viridis* are more heterogeneous and show intraspecific uncorrected divergence ranging from 1.2% (*Boophis viridis*) to 2.2% (*Boophis rappiodes*), in the 16S rRNA gene analysed fragment (Table 2). The species showing the highest values of intraspecific divergences is *Boophis rappiodes* (2.2%). This value of intraspecific divergence is computed based on the analyses of sequences coming from Andasibe and Ranomafana, two sites that are about 300 km far apart. The population of *Boophis ankarafensis* is about 400 km apart from the closest population of *Boophis bottae* (at Betampona) and their genetic distance is of 4.9%. As shown in Table 2, available values of intraspecific genetic divergence values never reach the minimum value of genetic divergence observed between different species of the *Boophis rappiodes* group (comparison *Boophis ankarafensis*/*Boophis bottae*).

**Table 2.** Genetic divergence in the analysed 16S rRNA mitochondrial gene fragment of the *Boophis rappiodes* group (*p*-distance transformed into percent using the complete deletion option). Pairwise distances calculated for intra- (in bold) and inter-specific genetic divergence. n.c. (not calculated).

	***Boophis ankarafensis***	***Boophis bottae***	***Boophis rappiodes***	***Boophis erythrodactylus***	***Boophis tasymena***	***Boophis viridis***
***Boophis ankarafensis***	**0**					
***Boophis bottae***	4.9%	**1.4%**				
***Boophis rappiodes***	9.0%	8.0%	**2.2%**			
***Boophis erythrodactylus***	11.4%	11.6%	11.9%	**n.c.**		
***Boophis tasymena***	9.9%	9.6%	9.7%	9.1%	**1.3%**	
***Boophis viridis***	11.1%	10.2%	12.0%	13.0%	12.1%	**1.2%**

The genetic distance between *Boophis ankarafensis* and the five other species of the *Boophis rappiodes* group ranges between 4.9% (comparison between *Boophis ankarafensis* and *Boophis bottae*) and 11.4% (comparison between *Boophis ankarafensis* and *Boophis erythrodactylus*). Among the species of the analysed species group the smallest genetic distance is observed between the newly described species and *Boophis bottae* (4.9%) and the highest value between *Boophis erythrodactylus* and *Boophis viridis* (13.0%). More details on genetic distances between species of the *Boophis rappiodes* group are provided in Table 2.

#### Advertisement call and acoustic monitoring.

**Figure 4.**
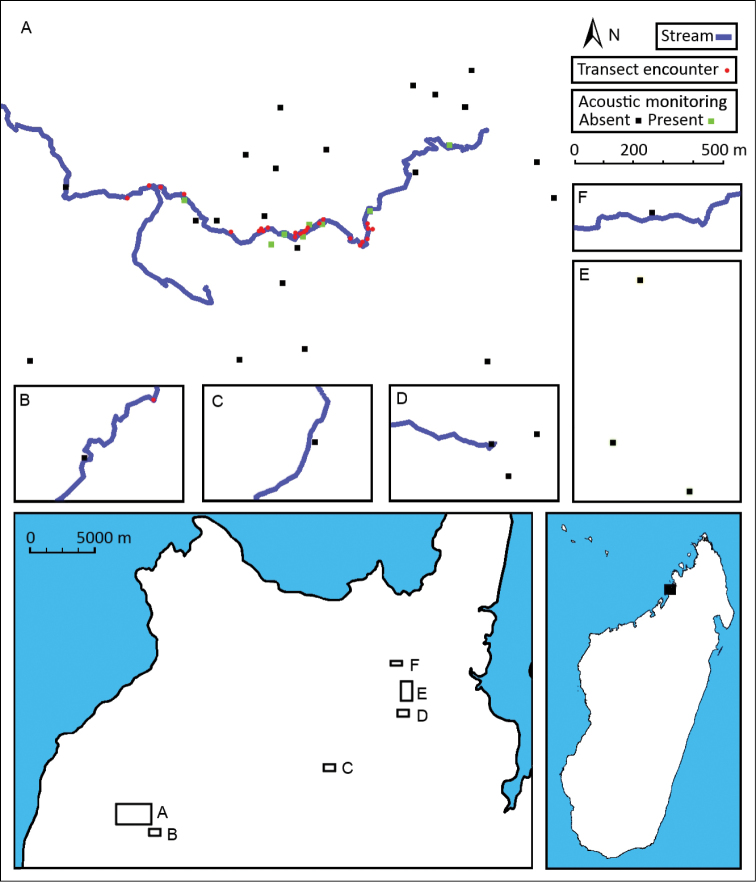
Detection of *Boophis ankarafensis* sp. n. Upper panel: Location of acoustic recording sites indicating presence (green) or absence (black) of vocal activity and transect encounters (red). Lower left: The Sahamalaza Peninsula: **A–B** Ankarafa Forest **C** Antafiabe Village **D–F** Anabohazo Forest. Lower right: location of the Sahamalaza Peninsula, in northwestern Madagascar.

**Figure 5.**
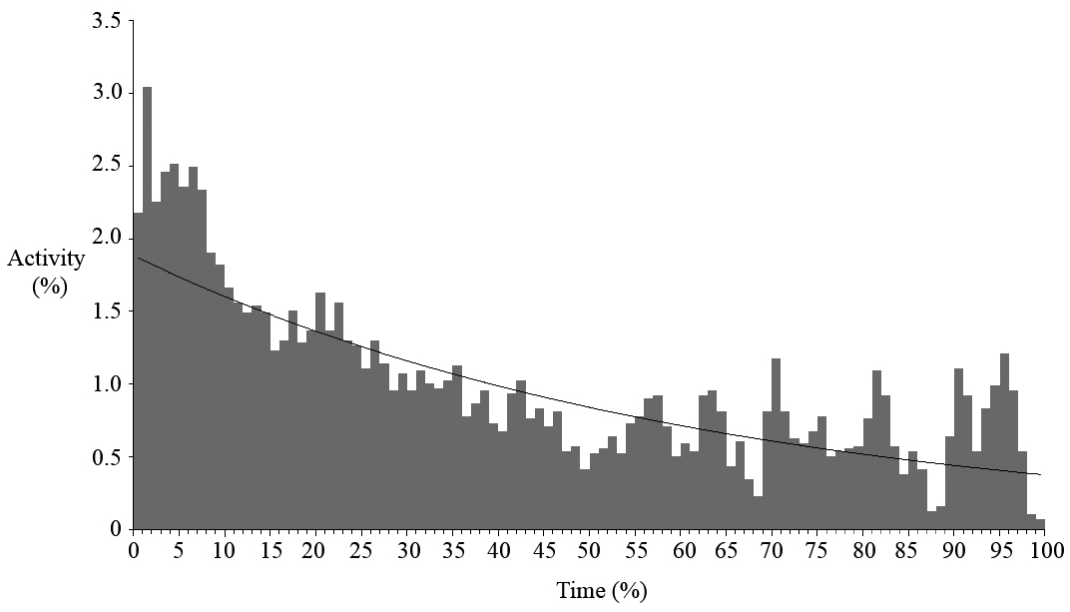
Nocturnal variation in calling activity of *Boophis ankarafensis* sp. n.; activity shown as a proportion of total calling activity and time shown as a proportion of night length from dusk [0%] until dawn [100%].

Vocalisations of *Boophis ankarafensis* sp. n. were recorded on thirteen occasions between October 2011 and January 2012. No males were found in calling activity between January and February 2013 (later in the wet season). Calls were detected from just 8 of the 37 locations monitored on the peninsula (Fig. 4). All eight of these locations were along the banks of a single stream in Ankarafa Forest. Vocalisations were detected within 3978 minutes of acoustic recordings and averaged 306 ± 181 minutes per night (n = 13). A total of 59.8% of these minutes contained vocalisations emitted by a single male caller, with the remainder emitted as a chorus of two or more callers. Total activity equalled 5576 minutes and average per night activity amounted to 429 ± 273 minutes (n = 13). Levels of calling fluctuated throughout the night (Fig. 5); there was a peak in activity just after dusk, followed by a slow decline until dawn, when the fewest vocalisations were detected (y = 0.019e^-0.015^x; *R*^2^ = 0.582).

**Figure 6.**
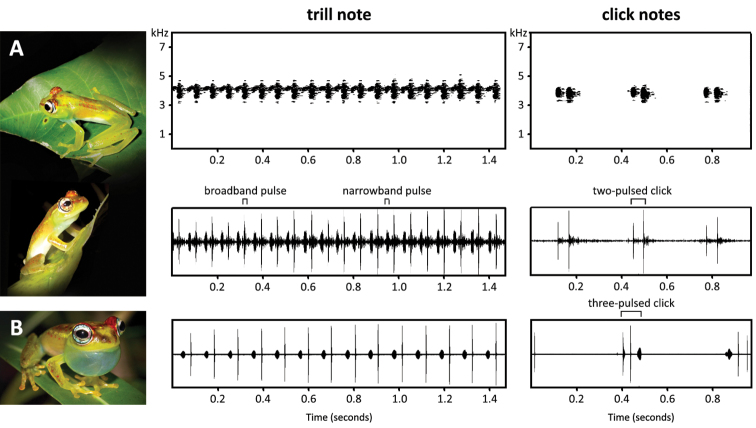
Representative call of *Boophis ankarafensis* sp. n. and comparative call of *Boophis bottae* from Betampona (Rosa et al. 2011, track #08): **A** *Boophis ankarafensis* sp. n. sonagrams (top) and oscillograms (bottom) referring to a section of trill note (type 1) constructed of alternating broad- and narrow-band pulses and click notes (type 2) (recorded at 25.2 °C, 13 October 2011) **B** *Boophis bottae* oscillograms of types 1 and 2 notes (recorded at 23.0 °C, 17 November 2007). Spectrogram parameters: FFT length 512, Hamming window.

The acoustic repertoire consists of two note types: a multi-pulsed trill (type 1; Fig. 6A) and a 1-3 pulsed click (type 2; Fig. 6B). Only 11 of the 13 occasions produced recordings of a sufficient audio quality to perform spectral analysis. Measurements were taken from five representative notes of each call type per night, thus a total of 55 trill notes and 55 click notes were measured (Table 3). From a subset of five trill notes, acoustic parameters were measured for ten sequential broadband pulses and ten sequential narrowband pulses per note. The environmental temperature of the recorded trill notes ranged between 25.2 and 30.3 °C (27.4 ± 1.08 °C, n = 55) and of the click notes between 22.9 and 29.7 °C (26.7 ± 1.41 °C, n = 55).

**Table 3.** Acoustic measurements of two note types of *Boophis ankarafensis*. In note type two durations are shown for the final two pulses.

Note	Parameter	Section	Mean±SD	Range
**Type 1. Trill**	**Duration**	Entire Note (s)	4.29 ± 1.29	1.61–8.09
		Broadband Pulse (ms)	3.47 ± 0.485	2.50–5.06
		Narrowband Pulse (ms)	17.9 ± 2.56	11.2–25.1
	**Pulse Rate**	20–30 Broadband Pulses (pulses/s)	15.0 ± 1.19	11.7–17.3
	**Peak Frequency**	40–60 Pulses (kHz)	4.14 ± 0.131	3.69–4.48
		Broadband Pulse (kHz)	4.19 ± 0.122	3.91–4.34
		Narrowband Pulse (kHz)	4.15 ± 0.108	4.00–4.31
**Type 2. Click**	**Duration**	Entire Note (ms)	50.9 ± 5.20	40.0–68.7
		Pulse one (ms)	6.37 ± 3.04	2.18–15.7
		Pulse two (ms)	6.61 ± 2.99	1.75–15.9
		Interpulse Interval (ms)	37.9 ± 6.28	20.0–50.7
	**Peak Frequency**	Entire Note (ms)	3.95 ± 0.162	3.57–4.30

The trill note measured 4.29 ± 1.29 s (1.614–8.091 s, n = 55) and is composed of alternating broadband and narrowband pulses. These notes had a broadband pulse rate of 15.0 ± 1.19 pulses/s (11.7–17.3 pulses/s, n = 25) and peak frequency of 4.14 ± 0.131 kHz (3.69–4.48 kHz, n = 55). Broadband pulses measured 3.47 ± 0.485 ms (2.50–5.06 ms, n = 50) whereas narrowband pulses were slightly longer at 17.9 ± 2.56 ms (11.2–25.1 m; n = 50). The broadband pulse had a peak frequency of 4.19 ± 0.122 kHz (3.91–4.34 kHz; n = 50), whereas the narrowband pulse measured 4.15 ± 0.108 kHz (4.00–4.31 kHz, n = 50). Bandwidths were 0.288 ± 0.169 kHz (0.120–0.780 kHz, n = 50) for the broadband pulse compared to just 0.155 ± 0.036 kHz (0.129–0.280 kHz, n = 50) for the narrowband pulse when measured at a -10 dB threshold. The majority of click notes consisted of two pulses, although singular and triple pulses were also observed. Click notes had a total duration of 52.9 ± 5.20 ms (40.0–68.7 ms; n = 55) and peak frequency of 3.95 ± 0.162 kHz (3.57–4.30 kHz; n = 50). Bandwidth of the click note measured 0.538 ± 0.234 kHz (0.120–0.143 kHz) at a -10 dB threshold.

The advertisement call of *Boophis ankarafensis* sp. n. sounds similar to that of the morphologically similar *Boophis bottae*; with both species possessing a trill note and a click note in their acoustic repertoires (Vences et al. 2006; Rosa et al. 2011). However, a number of differences can be used to distinguish them. In our comparison with *Boophis bottae* we selected only those notes recorded at a similar ambient temperature, and we have also distinguished the two pulse types of the trill by their relative differences in bandwidth, rather than duration as used by Vences and Glaw (2002); the broadband pulse and narrowband pulse of *Boophis ankarafensis* sp. n. are equivalent to the shorter and longer duration pulses of *Boophis bottae* respectively.

The trill note (type 1) of *Boophis ankarafensis* has a faster broadband pulse rate than the trill of *Boophis bottae* (13.4–13.5 pulses/s, 25.2 °C versus 8.12–11.6 pulses/s, 23.0 °C). The click note (type 2) of *Boophis ankarafensis* sp. n. usually contains just two pulses, and only rarely consists of three, in contrast the click notes of *Boophis bottae* are usually three-pulsed. Although only slightly divergent, it is discernable that the spectral frequency of the click note is lower in *Boophis ankarafensis* sp. n than in *Boophis bottae* (3.75–3.84 kHz, 22.9 °C versus 4.23–4.42 kHz, 23 °C). The trill note of *Boophis ankarafensis* shows a slightly lower broadband pulse peak frequency (4.10–4.24 kHz, 25.2 °C versus 4.30–4.64 kHz, 23 °C) but a similar narrowband pulse peak frequency (4.18–4.31 kHz, 25.2 °C versus 4.23–4.42 kHz, 23 °C) in comparison to *Boophis bottae*.

#### Ecology and natural history.

A total of 54 individuals of *Boophis ankarafensis* sp. n. were encountered between 29 October 2011 and 05 January 2012. An additional amplexing couple was found on 26 January 2013. Individuals were found at night in Ankarafa Forest along the banks of two streams; these streams became fast flowing after heavy rains during the wet season. No individuals were detected from sections of stream in open habitat where vegetation was absent, and so it should be considered a forest species.

Of the 56 encounters, 48 frogs were male and 8 female. Males of this species were found calling from vegetation approximately 0.5 to 2 m high. Vocalising males were often within close proximity to one another, positioned on different leaves of the same plant. All but one of the eight females were found in axillary amplexus with males (Fig. 3D–E); mating pairs were positioned on leaves overhanging the stream bank, and in one instance on a rock. Calling and breeding activity were most intense during the first few months of the rainy season (Oct–Dec), decreasing later in the season (Jan–Feb). A lone female was found in a tree during the day approximately 3 m high and 30 m from a stream.

#### Distribution, conservation and proposed IUCN status.

**Figure 7.**
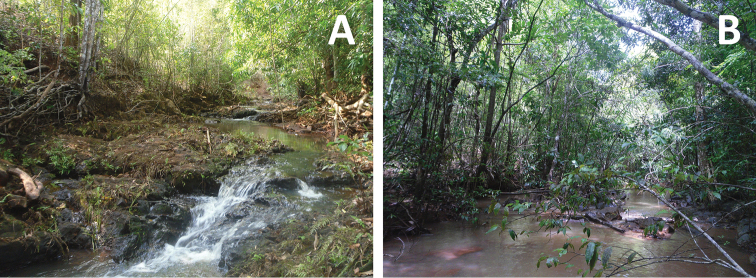
Habitat of *Boophis ankarafensis* sp. n. in Ankarafa Forest. **A** 21 November 2011; 14°23.39'S, 47°46.37'E; **B** 3 January 2012; 14°22.83'S, 47°45.57'E.

The suggested conservation status of this species was assessed using the criteria and guidelines of the IUCN Red List (IUCN Standards and Petitions Subcommittee 2011). This species is endemic to Madagascar and so far only recorded in a small forest fragment (Ankarafa Forest). Individuals were found along the vegetated banks of two streams, with forested sections of these streams extending for approximately 2 km of the more northerly stream (Fig. 7B), and just 1 km for the stream south of this (Fig. 7A). No individuals were found upstream of these two locations, where the streams are replaced by *Raphia*-dominated swampland or downstream where the stream-banks are cleared of vegetation. An average occurrence of 3 specimens per 200 m of river was determined from those transect searches where the specimens were detected (Table 4). This species was not detected from Anabohazo Forest, which aside from Ankarafa represents the only other large area of intact forest remaining on the peninsula. A survey of the Vavan’aneno River between Antafiabe and Ambinda villages, and a survey of Betsimpoaka village also failed to detect it.

**Table 4.** Count and frequency of *Boophis ankarafensis* sp. n., along sections of stream in Ankarafa Forest.

Date	Transect Location	No. of frogs	Transect Length (m)	Frequency /100 m
29/10/2011	Ankarafa North ‘upstream’	3	511	0.59
02/11/2011	Ankarafa North ‘midstream’	5	350	1.43
25/11/2011	Ankarafa South	3	814	0.37
26/11/2011	Ankarafa North ‘downstream’	7	609	1.15
15/12/2011	Ankarafa North ‘upstream’	18	511	3.52
29/12/2011	Ankarafa North ‘upstream’	11	511	2.15
05/01/2012	Ankarafa North ‘midstream’	7	350	2
**Total**		**54**	**3656**	**1.48**

If suitable habitat is considered to be all areas of Ankarafa Forest (likely an over-estimate) then this area totals less than 5 km^2^, giving an EOO (extent of occurrence) of less than 100 km^2^. If plots with a scale of 2 km^2^ are used to estimate AOO (area of occupancy), then this species occurs within 4 km^2^ of habitat, resulting in an AOO of less than 10 km^2^. Therefore the Critically Endangered thresholds for extent of occurrence and area of occupancy are both met (EOO < 100 km^2^ and AOO < 10 km^2^) (CR B1+2). The most serious threat to the species is habitat destruction through *tavy* practice (slash and burn agriculture), small-scale logging and the uncontrolled burning of neighbouring grasslands; a large out of control fire could easily affect the two subpopulations as they are separated by a distance of less than 2 km. Therefore, all individuals can be considered to occur within a single location only (CR B1a+2a). Given this on-going destruction of suitable habitat, population declines can be expected to continue unless some remedial action is taken (CR B1b(i, ii, iii, iv, v + B2b(i, ii, iii, iv, v)). Thus the species should qualify as Critically Endangered under criterion B (CR B1ab (i, ii, iii, iv, v)+2ab(i, ii, iii, iv, v) of the IUCN Red List (IUCN Standards and Petitions Subcommittee 2011).

## Discussion

*Boophis ankarafensis* sp. n. appears to be restricted to Ankarafa Forest on the Sahamalaza Peninsula in northwest Madagascar. The species represents the only member of the *Boophis rappiodes* group known from West Madagascar, and the only member to occur within a transitional forest type. The other members of the *Boophis rappiodes* group (*Boophis bottae*, *Boophis rappiodes*, *Boophis erythrodactylus*, *Boophis tasymena* and *Boophis viridis*) are confined to the eastern rainforest belt of Madagascar (Glaw and Vences 2007) aside for a single population of *Boophis erythrodactylus* known from Mahajeby Forest on the western slopes of Madagascar’s central figau (Vences and Glaw 2002).

The closest relative of *Boophis ankarafensis* is *Boophis bottae*, known from over 400 km to the east of Sahamalaza. It can be possible that in the past *Boophis bottae* has spread along the southern slopes of Madagascar’s northern massifs where it may have speciated and finally reached the transitional forest of the Sahamalaza Peninsula. Thus other populations of *Boophis ankarafensis* may be found here, although if they exist they will be isolated and fragmented due to habitat destruction. Other amphibian surveys in northwest Madagascar, e.g. in Sahamalaza, Manongarivo, Tsaratanana and Benavony, have failed to detect it, despite it possessing a conspicuous and distinctive advertisement call, (Vences and Glaw 2007, Raselimanana 2008) and so the species may be locally endemic to the peninsula. With this description, a total of three amphibian species are now known solely from Sahamalaza, with the species *Boophis tsilomaro* and *Cophyla berara* discovered during a previous survey in 2000 (Andreone et al. 2001; Vences et al. 2005, 2010). Thus, at just 26,000 ha, the peninsula appears to support a high level of amphibian endemicity, although further surveys of northwest Madagascar should be undertaken to search for additional populations.

All breeding behaviour of *Boophis ankarafensis* sp. n. was observed during the wet season along the banks of fast-flowing streams, indicating that its spawn is most likely laid in or adjacent to bodies of lotic water; a feature in common with most members of the *Boophis* subgenus (Glaw and Vences 2007). The streams in Ankarafa Forest flow throughout the year, in comparison all streams in Anabohazo Forest are seasonal. The species’ absence from this latter locality may be linked to the lack of perennial streams, for example its larval stage may not have enough time to complete development.

**Figure 8.**
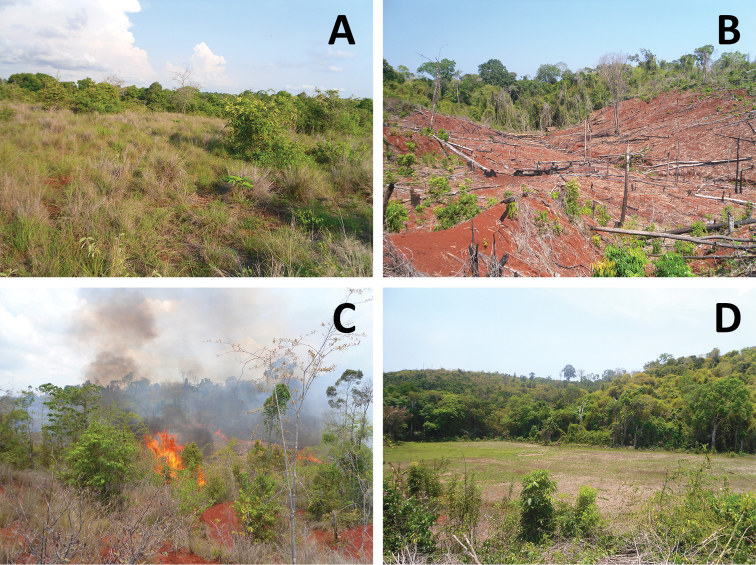
Anthropogenic disturbance within Ankarafa Forest: **A** Area of savannah, dividing Ankarafa Forest into many smaller fragments (14 December 2011; 14°22.77'S, 47°45.58'E) **B** Recent forest clearance (11 November 2011; 14°23.09'S, 47°44.92'E) **C** A fire lit to clear forest for agriculture (16 November 2011; 14°23.20'S, 47°44.80'E) **D** a *Tavy* field with intact forest in the background and the river acting as the boundary line (30 October 2011; 14°22.82'S, 47°45.28'E).

The frog was only found within intact forest and appears sensitive to anthropogenic disturbance. Intact forest is rare across the peninsula and aside from Anabohazo, where the species was not found, Ankarafa represents the largest area of remaining forest. Isolated populations of *Boophis ankarafensis* sp. n. may survive in residual pockets of gallery forest elsewhere on the peninsula, but Ankarafa is likely to harbour the largest and most sustainable population of this species. Their habitat in Ankarafa is fragmented and the two streams it is known from are separated by savannah (Fig. 8A). As *Boophis ankarafensis* sp. n. is arboreal, this will most likely limit gene flow between the two populations, potentially reducing its long-term viability.

Despite its protected status, Ankarafa is experiencing widespread deforestation (Seiler et al. 2012), furthermore much of this destruction is concentrated on the streamside forests which this species relies upon (pers. obs.). *Boophis ankarafensis* sp. n. is highly threatened and we propose that it should be classified as Critically Endangered (CR) according to the IUCN Red List criteria. The other apparent endemics face similar threats; *Cophyla berara* is already listed as CR (Andreone and Vences 2008), while *Boophis tsilomaro* most likely qualifies for CR status (Vences et al. 2010). A halt to all forest destruction and agricultural practices within the park must occur immediately to stop any further decline of Sahamalaza’s endemic amphibian fauna, or risk their possible extinction within the near future.

## Supplementary Material

XML Treatment for Boophis ankarafensis

## Supplementary material

Supplementary material 1 Calls of *Boophis ankarafensis* sp. n. emitted from streamside vegetation in Ankarafa Forest. doi: 10.3897/zookeys.435.7383.app1 Data type: audio file Explanation note: Calls of *Boophis ankarafensis* sp. n. emitted from streamside vegetation in Ankarafa Forest (Sahamalaza – Iles Radama National Park). Calls consist of multi-pulsed trill notes (type 1) and 1-3 pulsed click notes (type 2). Cut 1: chorus recorded on 13 October 2011, 18:34 h, 26.7 °C; Cut 2: chorus with background calls of *Cophyla berara* recorded on 30 December 2011 at 18:44 h, 28.4 °C. This dataset is made available under the Open Database License (http://opendatacommons.org/licenses/odbl/1.0/). The Open Database License (ODbL) is a license agreement intended to allow users to freely share, modify, and use this Dataset while maintaining this same freedom for others, provided that the original source and author(s) are credited.Samuel Penny, Franco Andreone, Angelica Crottini, Marc Holderied, Lovasoa Rakotozafy, Christoph Schwitzer, Gonçalo Rosa
